# The fibroblast growth factor system in cognitive disorders and dementia

**DOI:** 10.3389/fnins.2023.1136266

**Published:** 2023-05-05

**Authors:** Wujianwen Zhai, Tong Zhang, Yujing Jin, Shijing Huang, Manman Xu, Juhua Pan

**Affiliations:** Traditional Chinese Medicine Research and Development Center, Guang' Anmen Hospital, China Academy of Chinese Medical Sciences, Beijing, China

**Keywords:** fibroblast growth factor, fibroblast growth factor receptor, dementia, cognitive disorders, Alzheimer’s disease, neurodegenerative diseases

## Abstract

Cognitive impairment is the core precursor to dementia and other cognitive disorders. Current hypotheses suggest that they share a common pathological basis, such as inflammation, restricted neurogenesis, neuroendocrine disorders, and the destruction of neurovascular units. Fibroblast growth factors (FGFs) are cell growth factors that play essential roles in various pathophysiological processes via paracrine or autocrine pathways. This system consists of FGFs and their receptors (FGFRs), which may hold tremendous potential to become a new biological marker in the diagnosis of dementia and other cognitive disorders, and serve as a potential target for drug development against dementia and cognitive function impairment. Here, we review the available evidence detailing the relevant pathways mediated by multiple FGFs and FGFRs, and recent studies examining their role in the pathogenesis and treatment of cognitive disorders and dementia.

## 1. Introduction

Dementia which occurs in the late stages of the cognitive disorders is one of the most prevalent mental illnesses, particularly among the elderly (Bennett and Thomas, 2014). The primary clinical manifestation of cognitive disorders is a decline in cognitive function, accompanied by disordered thinking, memory deficits, sensory disturbances, and poor concentration, which are risk factors and precursor symptoms of dementia, including Alzheimer’s disease (AD; Anderson, 2019), vascular dementia (VaD; Dichgans and Leys, 2017), and Huntington’s disease (HD; Papoutsi et al., 2014; van der Flier et al., 2018; Iadecola et al., 2019). Recent research has shown that the pathogenesis of those diseases is associated with various pathological mechanisms, including neuroinflammation (Byers and Yaffe, 2011; Leng and Edison, 2021), oxidative stress (Luca and Luca, 2019; Manduca et al., 2020), immune dysregulation (Hayley et al., 2021), disruption of neurotransmitters (Singh, 2009), synaptic plasticity injury (Byers and Yaffe, 2011; Skaper et al., 2017), and neuroendocrine disorders (Manduca et al., 2020). FGFs are cell growth factors involved in multiple critical pathophysiologic processes in the human body via paracrine and autocrine pathways such as embryonic development and angiogenesis, neurogenesis, wound healing, and glucolipid metabolism, which need to bind fibroblast growth factors receptor (FGFR) to produce physiological effects (Hui et al., 2018). Some researchers believe that FGFs have the potential to become a novel biological marker for the diagnosis and prognosis of neurodegenerative diseases, and these results may also indicate new targets for treatment (Galvez-Contreras et al., 2016). This review focuses on how the FGF-FGFR system changes and affects the pathogenesis and treatment of cognitive disorders and other dementia. We hypothesized that maintaining a dynamic balance in the FGF-FGFR system would be beneficial for nerve repair and neuroprotection to reduce the clinical symptoms and risk of cognitive disorders.

## 2. Fibroblast growth factors in cognitive disorders and dementia

Patients with cognitive impairment without meeting the diagnostic criteria for dementia tend to be diagnosed by clinicians as mild cognitive impairment (MCI), while patients with concomitant cerebrovascular pathological changes or whose cognitive deficit occurs secondary to cerebrovascular disease can be diagnosed as vascular cognitive impairment. Cognitive impairment can be classified as ‘functional’ or ‘non-functional’ depending on the presence or absence of neurodegenerative changes (Ball et al., 2020). For the former, only cognitive, memory, and emotional impairments occur, which are less likely to deteriorate into dementia; for the latter, the subjective cognitive decline is accompanied by structural changes in the brain and eventually degenerates into dementia.

The pathological changes of dementia are, on the one hand, neurodegenerative changes due to senescence, such as AD, HD, frontotemporal dementia, and Parkinson’s disease dementia; on the other hand, dementia is also closely associated with cerebrovascular injury, metabolic disorder, intracranial infections, intracranial tumors, hypoxic–ischemic brain injury, which secondary to stroke, atherosclerosis, chronic renal insufficiency, diabetes, hyperlipidemia, hypertension, and other diseases. These suggest a mixed pathology for dementia. Cellular senescence apoptosis and abnormal autophagy, inflammation, oxidative stress, vascular damage, and metabolic dysfunction interact to induce neuropathic protein accumulation and morphological changes in the brain, which are further disordered in a vicious cycle of neurodegenerative disease (Gonzales et al., 2022).

The FGF-FGFR system consists of seven receptors and 22 ligands that are required to bind to their corresponding receptors via acetyl heparan sulfate (HS) cofactor or α/β-Klotho (α/β-KL) transmembrane proteins to exert their physiological effects (Beenken and Mohammadi, 2009; Xie et al., 2020). The 22 FGFs can be divided into different subgroups based on sequence homology and developmental characteristics. For example, FGF-15/19, FGF-21, and FGF-23 are members of a particular FGF type called endocrine FGFs, which require KL proteins to bind to their receptors because of their low affinity for HS (Belov and Mohammadi, 2013; Owen et al., 2015; Hui et al., 2018cc Deng et al., 2019; Chen K. et al., 2022). Except for homologous factor subfamily (FGF-11/12/13/14) and endocrine FGF subfamily, other FGFs all belong to paracrine subfamilies, including FGF-1, FGF-2, FGF-9, and FGF-17 (Deng et al., 2019). Abnormal FGF expression levels have been observed by researchers in patients with dementia and other cognitive disorders (Stopa et al., 1990; Mashayekhi et al., 2010; Hensel et al., 2016; Liang et al., 2021). Moreover, the effects of FGFs on neuromodulation and cognitive improvement have been validated in literature (Lee et al., 2011; Taliyan et al., 2019). Fibroblast growth factors in cognitive disorders and dementia is summarized in Table 1.

**Table 1:** The expressions and prospects of the FGF/FGFR system in cognitive disorders and dementia.

Members	Receptors	Sample	Trends	Main effects	Prospects	References
FGF-1	FGFR-1b,1c	Cortex homogenate (AD)	↑	Reduced inflammation and oxidative stress, repaired BBB, suppressed excitotoxicity, improved insulin sensitivity	AD, VaD, MCI, VCI	Takami et al. (1998), Thorns et al. (2001), Yamagata et al. (2004), Mashayekhi et al. (2010), Tsai et al. (2015), Wu et al. (2017), and Liang et al. (2021)
	FGFR-2b,2c	Serum (AD)	↓			
	FGFR-3b,3c	CSF (AD)	↓			
	FGFR-4					
FGF-2	FGFR-1b,1c	Cortex homogenate (AD)	↑	Promoted neurogenesis, inhibited neurotoxicity, extended neurons life-span, induced angiogenesis, inhibited inflammation	AD, VaD, HD	Jin et al. (2005), Duff et al. (2010), Wang et al. (2016), Tyebji and Hannan (2017), Liu M. et al. (2018), and Ilieva et al. (2019)
	FGFR-2c					
	FGFR-3c	Serum (AD)	↓			
	FGFR-4					
FGF-9	FGFR-2c	Hippocampus homogenate (AD)	↑	Promoted neuronal development, inhibited oxidative stress, suppressed apoptosis, promoted neurogenesis	AD, HD	Nakamura et al. (1998), Chuang et al. (2015), and Yusuf et al. (2019, 2021a,b)
	FGFR-3b,3c	Serum (HD)	↓			
FGF-17	β-KL/FGFR-2	N/A	N/A	Supported oligodendrocyte precursor cell growth, inhibited FGF-19 pathway	MCI	Liu S. et al. (2018) and Iram et al. (2022)
	FGFR-3c					
FGF-15/19	β-KL/FGFR-4	N/A	N/A	Inhibited HPA axis hyperexcitability, reduced insulin resistance, regulated neurotransmitter homeostasis, promoted neurogenesis, regulated bile acid metabolism	AD, MCI	Marcelin et al. (2014), McMillin et al. (2015), Perry et al. (2015), Mertens et al. (2017), Liu S. et al. (2018), and Li et al. (2020)
FGF-21	β-KL/FGFR-1	Serum (AD)	↓	Improved BBB integrity, repair cerebrovascular endothelium, inhibited inflammation, promoted neurogenesis, suppressed apoptosis, maintained neurotransmitter homeostasis, regulated lipid metabolism and glucose metabolism, enhanced insulin sensitivity	AD, VaD	Sa-Nguanmoo et al. (2016), Kuroda et al. (2017), Chen et al. (2018), Zheng et al. (2019), Jiang et al. (2020), and Wang et al. (2020)
	PPAR-γ					
FGF-23	α-KL/FGFR-1c	Serum (MCI)	↑	Regulated phosphate homeostasis and glucose metabolism, promoted neurogenesis	MCI	Liu et al. (2011), Drew et al. (2014), and Drew and Weiner (2014)
	FGFR-2c					
	FGFR-3c					

AD, Alzheimer’s disease; BBB, blood–brain barrier; CSF, cerebrospinal fluid; FGF, fibroblast growth factor; FGFR, fibroblast growth factor receptor; HD, Huntington’s dementia; MCI, mild cognitive impairment; VCI, vascular cognitive impairment; VaD, vascular dementia; α/β-KL, α/β-klotho protein.

### 2.1. Paracrine fibroblast growth factors

#### 2.1.1. FGF-1

Fibroblast growth factor-1 (FGF-1), also known as acidic fibroblast growth factor (aFGF), is secreted by meningeal cells in the ventricles of the third ventricle and is widespread in multiple tissues, including the hippocampus, pituitary gland, heart, and kidney (Beenken and Mohammadi, 2009; Mashayekhi et al., 2010). FGF-1 can modulate physiological activities such as embryonic development, angiogenesis, cell proliferation and differentiation, and adult hippocampal neurogenesis (AHN) by activating different FGFRs (Lee et al., 2011; Tsai et al., 2015; Gasser et al., 2017; Sun et al., 2021). Although there remains a paucity of studies investigating the specific role of FGF-1 in the pathological mechanisms of cognitive impairment, several lines of evidence have shown that dysregulation of FGF-1 expression is closely associated with dementia, especially Alzheimer’s disease (Takami et al., 1998; Yamagata et al., 2004).

AHN refers to the process by which neural stem cells in the hippocampus undergo symmetric or asymmetric division into neuroblasts, gradually migrate to specific regions after cell proliferation and then differentiate into new neurons and other resident brain cells. Thus, AHN is the fundamental physiological basis for neuroplasticity. After binding to FGFR-1, FGF-1 has a significant reparatory effect on damaged neurons in the cortex and hippocampus, reducing inflammatory factors secreted by microglial (MG) activation, thereby promoting neurogenesis and vascular regeneration (Tsai et al., 2015). However, there is no one-to-one correspondence between the FGFs and FGFRs. For instance, FGF-1 secreted by activated astrocytes (ASTs) induces neuroinflammation instead of neuroprotection if by binding to FGFR-2 (Lee et al., 2011). The neurovascular unit (NVU) is a microstructure composed of neurons, neuroglia, the BBB, and the extracellular matrix, and plays an essential role in maintaining nerve function. The BBB is an essential barrier, that protects the stability of the neural microenvironment and is the core of NVU coupling, providing protection against leakage of neurotoxic substances from the blood into the cerebral parenchyma. The BBB consists of cerebral microvascular endothelial cells and is connected to astrocytes, pericytes, perivascular macrophages, and basement membranes, which are hyperpermeable in depressed patients, triggering inflammation of the central nervous system (CNS) and endothelial damage to cerebral microvessels, further exacerbating neuronal injury. At the same time, FGF-1 could repair the BBB by upregulating the expression of tight junction proteins and adherens junction proteins via activating p-PI3K, PI3K, p-Akt, and Akt to suppress RhoA but activate Rac1 (Wu et al., 2017). Preliminary observations suggest that FGF-1 expression is reduced in neurons of the internal olfactory cortex in patients with AD, which inhibits the expression of calcium-binding proteins and induces overexpression of the N-methyl-D-aspartic acid receptor (NMDAR), resulting in the disruption of calcium homeostasis and glutamate-mediated excitotoxicity (Thorns and Masliah, 1999; Thorns et al., 2001). Several recent studies reported a significant increase in FGF-1 levels in both plasma and cerebrospinal fluid in AD (Mashayekhi et al., 2010; Liang et al., 2021), suggesting that there may be differences in local concentrations of FGF-1, but this remains to be verified further.

**Figure 1:**
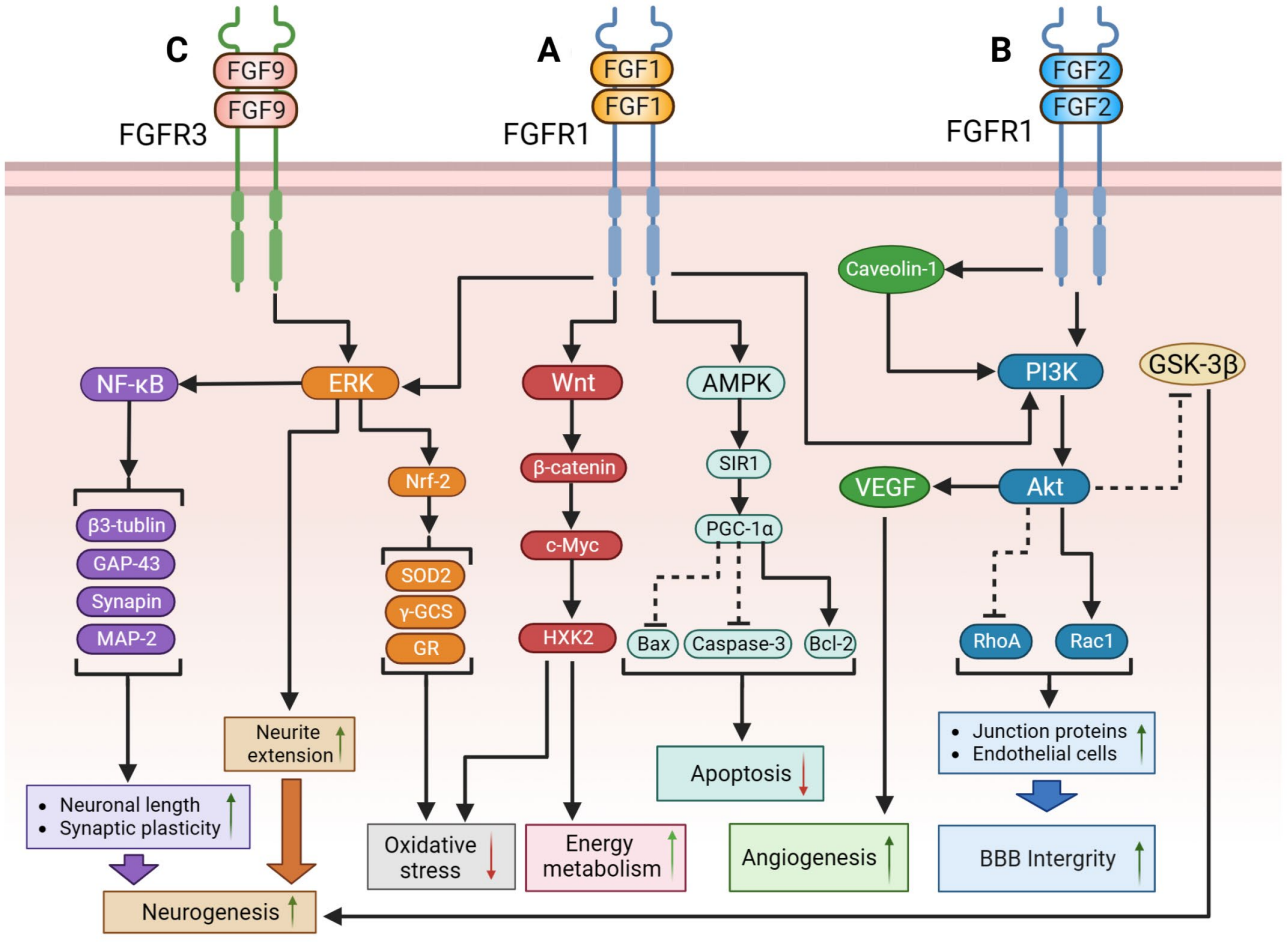
The potential therapeutic mechanism of paracrine FGFs (FGF-1, FGF-2, FGF-9). **(A)** FGF-1might regulate the following signaling pathways to improve cognition function: (1) FGF-1 activates PI3K/Akt pathway to suppress RhoA but activate Rac1, causing the upregulation of BBB integrity; (2) FGF-1 activates ERK pathway to extend neurite for promoting neurogenesis; (3) FGF-1 activates Wnt/β-catenin/c-Myc/ HXK2 pathway to improve mitochondrial energy metabolism and suppress oxidative stress; (4) FGF-1 exert the activation of the AMPK/SIRT1/PGC-1α pathway to modulate the apoptosis. **(B)** (1) FGF-2 might inhibit GSK-3β through PI3K/AKT pathway to promote adult hippocampal neurogenesis; FGF-2 could also repair blood–brain barrier integrity through the activation of PI3K/Akt/Rac1axis, but inhibiting RhoA; (2) Additionally, protect astrocytes and induce angiogenesis to improve cerebral blood supply by upregulating the Caveolin-1/PI3K/AKT/VEGF pathway. **(C)** FGF-9 might activate ERK/Nrf-2pathway to suppress oxidative stress and activate the ERK/NF-κB pathway to improve hippocampal neurogenesis. The solid black lines and arrows indicate the activation of the signaling pathway, and the dashed black lines and T-arrows indicate the inhibition of the signaling pathway. FGF, fibroblast growth factor; PI3K, phosphatidylinositol-3-hydroxy kinase; AKT, protein kinase B; Rac1, rac family small GTPase 1; RhoA, ras homolog family member A; BBB, blood–brain barrier; ERK, extracellular signal-regulated kinase; HXK2, hexokinase 2; AMPK, adenosine monophosphate activated protein kinase; SIR1, sirtuin1; PGC-1α, peroxisome proliferator-activated receptors-γ coactivator 1-α; GSK-3β, glycogen synthase kinase-3β; VEGF, vascular endothelial growth factor; Nrf-2, nuclear factor erythroid-like 2; SOD2, superoxide dismutase 2; γ-GCS, gamma-glutamylcysteine synthetase; GR, glutathione reductase; NF-κB, nuclear factor kappa-B; MAP-2, microtubule-associated protein-2; GAP-43, growth association protein-43.

Glucose is the primary energy source of the brain (Mergenthaler et al., 2013). In addition to providing energy for neural activity, glucose indirectly regulates the transmission of signals that affect neural function (Dienel, 2012; Hossain et al., 2020). Cerebral glucose metabolism plays a crucial role in the physiological mechanisms of cognitive function and the pathomechanisms of dementia. Therefore, cerebral insulin resistance can trigger oxidative stress, mitochondrial dysfunction, inflammation, and autophagy by inducing severe dysfunction of extracellular glucose transport and intracellular glucose metabolism disorders, leading to neurodegeneration (Chen and Zhong, 2013; Butterfield and Halliwell, 2019). Notably, patients with AD or MCI exhibit a dramatic decline in cerebral glucose metabolism due to insulin resistance (Chen and Zhong, 2013; Butterfield and Halliwell, 2019). Researchers have also found that patients with dementia have significant insulin resistance in their hippocampi (Wijesekara et al., 2018; Hamer et al., 2019), suggesting that dysregulation of brain insulin signaling pathways may play an important role in cognitive dysfunction. Exogenous FGF-1 has been shown to reduce blood glucose concentrations and increase insulin sensitivity (Holmes, 2014; Suh et al., 2014; Gasser et al., 2017; Tennant et al., 2019) via modulating the Wnt/β-catenin (Sun et al., 2021) and AMPK pathways (Chen Q. et al., 2022). Thus, FGF-1 could indirectly ameliorate cognitive impairment by attenuating insulin resistance in local brain regions. The potential therapeutic mechanisms of FGF-1 are shown in Figure 1.

#### 2.1.2. FGF-2

FGF-2, also known as basic fibroblast growth factor (bFGF), was the first fibroblast growth factor to ever be identified. In the CNS, FGF-2 is primarily produced by ASTs and distributed throughout the cortex, hippocampus, and hypothalamus. Physiological concentrations of FGF-2 have been reported to promote proliferation, differentiation, migration, and maturation of neural stem cells and neuroglia, maintain cortical synaptic connectivity, stimulate neurogenesis, inhibit neuroinflammation, and exert tremendous neuroprotective and neurotrophic effects (Numakawa et al., 2015; Wu et al., 2017). The expression levels of FGF-2 are elevated in a compensatory manner in dementia (Stopa et al., 1990; Tennakoon et al., 2022), whereas the artificial modulation of FGF-2 levels improves cognitive function in rodents (Kiyota et al., 2011; Feng et al., 2012; Numakawa et al., 2015). In addition, FGF-2 has been shown to be a significant therapeutic target for certain anti-dementia medications, such as memantine (Namba et al., 2010).

Hippocampal neuronal cells are critical for the regulation of memory, learning, cognition, emotion, and other functions. FGF-2 has been proposed to stimulate the growth of neuronal synapses and regulate tight junction proteins in vascular endothelial cells via activating the PI3K/AKT pathway to promote AHN and protect the BBB (Wang et al., 2016; Ilieva et al., 2019). Neuroglia cells are a crucial component of the NVU, as they maintain the stability of the physiological functions of the neurons in the brain and consist of ASTs, MG, and oligodendrocytes (OLs). In HD, FGF-2 could promote the proliferation and recruitment of neuronal cells and inhibit the neurotoxicity produced by polyglutamine protein aggregation, which would prolong the lifespan of neurons (Jin et al., 2005; Duff et al., 2010; Tyebji and Hannan, 2017). ASTs, which have both an anti-inflammatory resting state and a pro-inflammatory reactive state, are the most abundant and largest neuroglia in the CNS and play a key role in neurotransmitter regulation and energy provision. In cognitive disorders, the density and number of resting ASTs decrease with the aggravation of symptoms, which leads to a decline in neurotrophic factors and nerve growth factors secreted by ASTs, while the number of reactive ASTs, which produce large amounts of inflammatory factors and neurotoxins is raised (Wang et al., 2017; Lu et al., 2020). Excessive activation of GSK-3β can induce hyperphosphorylation of tau. Furthermore, spatial memory deficits and cognitive decline in AD could be ameliorated by FGF-2 through a simultaneous decrease in amyloid-β (Aβ) and microtubule-associated protein tau while increasing the number of resting ASTs in the hippocampal dentate gyrus (Katsouri et al., 2015). A possible explanation for this result is that exogenous FGF-2 may suppress the over-activated GSK-3β-related pathway and strengthened neurogenesis (Hong et al., 2016). It could activate p-GSK-3β and GSK-3β, triggering tau hyperphosphorylation to aggravate cognitive decline, but could also inhibit GSK-3β via activating the Akt pathway to promote hippocampal neurogenesis. We assumed that these mixed results might attribute to different molecular weight isoforms of FGF-2 activating different downstream pathways. Moreover, FGF-2 could protect ASTs from ischemia–reperfusion injury, a common pathological basis of VaD, and induce angiogenesis to ameliorate chronic cerebral hypoperfusion by upregulating the Caveolin-1/PI3K/AKT/vascular endothelial growth factor (VEGF) signaling pathway (Liu M. et al., 2018).

Neuroinflammation is a critical pathological mechanism that disrupts NVU homeostasis. The levels of multiple pro-inflammatory factors observed in both the blood and cerebrospinal fluid of patients with dementia were higher than those in healthy individuals (Shen et al., 2019). For example, MG is an integral type of neuroglia for maintaining the AHN process with a pro-inflammatory phenotype M1 and an anti-inflammatory phenotype M2. Activation of M1 induces neuroinflammation, which inhibits FGF-2 expression and activates downstream ERK pathway in the hippocampal region, thereby restraining the AHN process. In contrast, exogenous FGF-2 could effectively inhibit interleukin-1β (IL-1β), interleukin-6 (IL-6), tumor growth factor-α (TNF-α) and other pro-inflammatory factors produced by M1-type MG in an attempt to improve depressive-like behaviors (Tang et al., 2017). FGF-2 may also enhance the activation of M2-type MG and its phagocytosis of Aβ, thereby promoting neurogenesis in dementia (Kiyota et al., 2011). OLs are crucial for the modulation of the cerebrovascular system and the maintenance of white matter structure and function, while FGF-2 can promoted proliferation, maturation, differentiation, and migration of OLs and suppress their apoptosis (Miyamoto et al., 2014). White matter hyperintensities (WMH) are the imaging features of white matter lesions, with several studies suggesting a clinically relevant link between WMH burden and cognitive decline in MCI and AD (Garnier-Crussard et al., 2022; Kamal et al., 2022). Researchers have also found that senescent oligodendrocyte precursor cells (OPCs) were the primary neuroglial cell expressed in neuritic plaques, which could induce cell senescence in AD, causing cognitive decline (Zhang et al., 2019). Therefore, modulation of neuroglial cells (OLs, ASTs, and MG) via the FGF-2 pathway may play a role in the treatment of cerebral white matter lesions and VaD via angiotensin-converting enzyme II (Wakayama et al., 2021). The current application of FGF-2 is mainly in synthetic recombinant human-derived FGF-2 which has several limitations such as short half-life in the blood, inability to completely cross the BBB, or side effects on the vascular system (Bogousslavsky et al., 2002). However, novel synthetic compounds such as SUN11602 have been proposed, which mimic the structure of FGF-2, and could potentially avoid the aforementioned defects and exert neuroprotective effects of FGF-2 to a certain extent (Ogino et al., 2014; Ardizzone et al., 2022).

In recent decades, several researchers have observed opposing trends in the changes of FGF-2 in serum and brain tissue in dementia. For example, Katsouri et al. (2015) found that the concentration of FGF-2 in the frontal cortical homogenates of patients with AD was decreased, rather than increased, contrary to previous studies (Stopa et al., 1990). Additionally, the original hypothesis assumed that FGF-2 attracts neurons into plaques in AD (Cummings et al., 1993); however, recent studies have argued that FGF-2 confers neuroprotective effects (Feng et al., 2012; Katsouri et al., 2015). We speculate that discrepancies could be explained with the following reasons: (1) Numerous cells in the peripheral system, such as blood cells, bone marrow stromal cells, and smooth muscle cells, can also secrete FGF-2. Therefore, serum FGF-2 levels might be affected by other systemic diseases in ways that truly reflect the level of FGF-2 in the CNS. (2) Subjects in the discussed studies were not receiving uniform treatment, which may increase the uptake of FGF-2 into damaged neuronal cells, resulting in a relative decrease in serum FGF-2 levels. (3) An increase in serum FGF-2 levels could also be a result of compensatory secretion to repair nerve injury. (4) There are at least six different molecular weight isoforms of FGF-2 (Chlebova et al., 2009), which may have different physiological roles, but routine detection could be unable to show the differences between the isomers (Jiang et al., 2007; Chen X. et al., 2019). Therefore, despite ample evidence supporting the key role of FGF-2 in cognitive impairment and dementia, it still fails to become an independent clinical diagnosis or assessment indicator. These points made above are also the common challenges we face during the study of the FGF family members. The potential therapeutic mechanisms of FGF-2 are shown in Figure 1.

#### 2.1.3. FGF-9

FGF-9 is synthesized by neurons of the CNS which binds to FGFR-2, FGFR-3, but not FGFR1 and FGFR4, playing a prominent role in angiogenesis, neurogenesis, cellular differentiation and cardiac development (Hecht et al., 1995; Reuss and von Bohlen und Halbach, 2003; Wang S. et al., 2018). Nakamura et al. (1998) found that high levels of FGF-9 expression in the hippocampus of patients with AD promoted the activation of the pro-inflammatory phenotype of ASTs around senile plaques, thereby exacerbating cognitive impairment. Alternatively the expression level in the CNS of FGF-9 may decrease in patients with HD. Previous results of Chuang et al. (2015) which have identified prominent FGF-9 and FGFR-3 expression in primary neuron-enriched cultures, suggesting the FGF-9 effects are cell-type specific in brain, may be a likely explanation for the different expression trends of FGF-9 in AD and HD.

It has been shown that exogenous FGF-9 may promote neuronal development and synaptic growth in striatal cell models of HD in response to anti-oxidant and anti-apoptotic effects via extracellular signaling that modulates the ERK/NF-κB pathway (Yusuf et al., 2019, 2021a,b), which provides a novel insight into the treatment of cognitive decline due to HD. FGF-9 activates Nrf-2 to upregulate transcription factors (SOD2, γ-GCS, and GR) for suppressing oxidative stress, through the activation of the ERK pathway. Additionally, FGF-9 could also activate the ERK/NF-κB pathway to upregulate β-tubulin, MAP-2, GAP-43, and synapsin to improve the neuronal length and synaptic plasticity. These studies suggest that FGF-9 overexpression in AD might be a compensatory mechanism for neuroprotection.

In addition, a negative correlation was found between FGF-9 and adiponectin (ADPN). ADPN is a pleiotropic adipocyte-secreting hormone with neurotrophic effects. Animals knocked out of the ADPN gene could present depressive-like behavior and cognitive deficits (You et al., 2021). Researchers have found that the Chinese herbal extract carnosic acid could simultaneously reduce FGF-9 levels and raise ADPN levels both in mice’s serum and hippocampal tissues, thereby ameliorating depression-like symptoms (Azhar et al., 2021; Wang et al., 2021). Interestingly, Carnosic acid could also alleviate AD-induced cognitive decline by inhibiting neuroinflammation, ameliorating cholinergic deficits, and enhancing energy metabolism (Chen Y. et al., 2022; Yi-Bin et al., 2022). We supposed that FGF-9 may also participate in carnosic acid’s anti-dementia mechanism of action, but it requires further study. The potential therapeutic mechanisms of FGF-9 are shown in Figure 1.

#### 2.1.4. FGF-17

**Figure 2:**
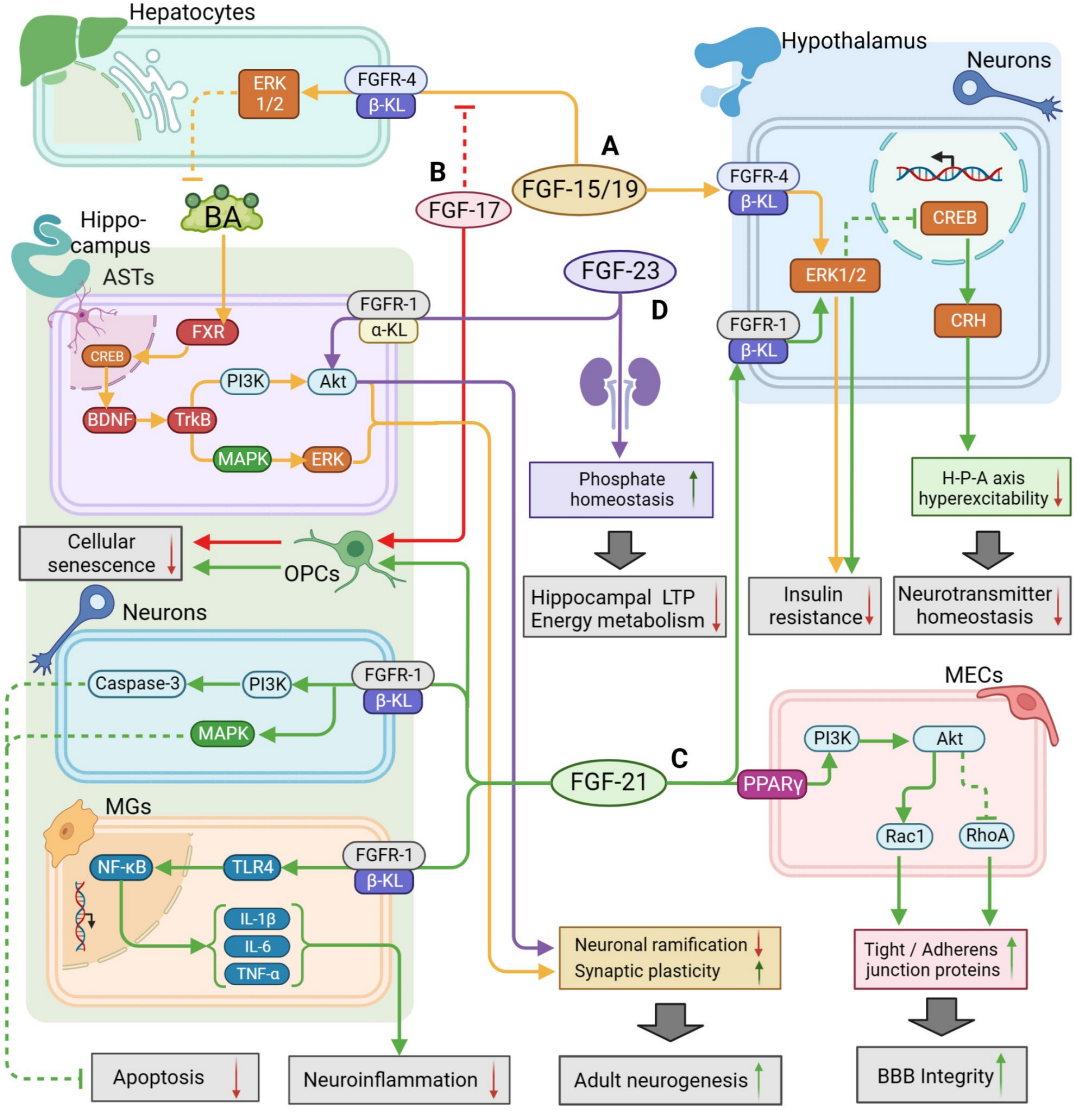
The potential therapeutic mechanism of endocrine FGFs (FGF15/19, FGF21, FGF23) and FGF17. **(A)** FGF-15/19/FGFR4/β-KL might activate the ERK1/2 pathway in hepatocytes to regulate BA metabolism for activating BDNF/TrkB/PI3K pathway and BDNF/TrkB/MAPK pathway in hippocampal ASTs, leading to improvement of adult neurogenesis; FGF15/19 could also penetrate the BBB to inhibit insulin resistance by activating the ERK1/2 pathway in hypothalamus. **(B)** FGF-17 could enhance the growth of OPCs to defer cell aging and suppress FGF-15/19 pathway as a competitive inhibitor. **(C)** FGF-21 could enhance BBB integrity by activating PI3K/Akt pathway in MECs, and reduce neuroinflammation through the suppression of TLR4/ NF-κB pathway in MGs; FGF-21 could also inhibit apoptosis by activating PI3K/Caspase-3 pathway and MAPK pathway in neurons; FGF-21 could also regulate HPA axis function by activating ERK/CREB pathway to maintain neurotransmitter balance; Additionally, FGF-21 might defer OPCs aging for repairing cognitive function. **(D)** FGF-23/FGFR-1/α-KL might directly activate the AKT pathway to inhibit neuronal ramification but maintain synaptic plasticity; FGF-23 expressed in the kidney could also indirectly regulate phosphate homeostasis to promote LTP and mitochondrial energy metabolism for improvement of cognitive function. The solid arrows indicate the activation of the signaling pathway, and the dashed T-arrows indicate the inhibition of the signaling pathway. The yellow lines represent the FGF-15/19 pathway, the red lines represent the FGF-17 pathway, the green lines represent the FGF-21 pathway and the purple lines represent the FGF-23 pathway. FGF, fibroblast growth factor; BA, bile acid; FXR, farnesoid X receptor; GR, glucocorticoid receptors; KL, klotho protein; BBB, blood–brain barrier; BDNF, Brain Derived Neurotrophic Factor; PI3K, phosphatidylinositol-3-hydroxy kinase; Akt, protein kinase B; TrkB, tyrosine kinase receptor B; ERK, extracellular signal-regulated kinase; MAPK, mitogen-activated protein kinase; CREB, cAMP-response element binding protein; TLR4, toll like receptor-4; NF-κB, nuclear factor kappa-B; OPCs, oligodendrocyte precursor cells; ASTs, astrocytes; MECs, microvascular endothelial cells; LTP, long-time potentiation.

FGF-17 is mainly expressed in the cerebrospinal fluid, plasma, and cortical neurons. It binds mainly to FGFR-3 to regulate downstream signaling, fulfilling vital roles in embryonic development, cell proliferation, and early neurogenesis, which declines during senescence (Hoshikawa et al., 1998; Machado et al., 2015; Han et al., 2019; Sathyan et al., 2020; Iram et al., 2022). Iram et al. (2022) recently observed that the growth of OPCs was suppressed after blocking the FGF-17/FGFR-3 pathway, which impaired hippocampal function in mice and provoked cognitive impairments and memory loss. Furthermore, they proved that exogenous injection of FGF-17 could enhance cognitive and memory performance in older mice. This finding is contrary to a previous study by Scearce-Levie et al. (2008) which found that young mice did not show obvious symptoms of cognitive impairment after knocking out the FGF-17 gene. These differences can be attributed to the fact that the subjects were in different physiological states that were influenced by their age. Besides, FGF-17 has been found to bind to the β-KL/FGFR-2 homodimer in the hypothalamus and show antagonize FGF-15/19, which blocked the insulin signaling pathway regulated by FGF-15/19 and triggered a decrease in glucose tolerance (Liu S. et al., 2018). The following may indicate that FGF-17 exerts distinct effects when bound to different receptors separately in the hippocampus or hypothalamus. Therefore, the therapeutic potential and side effects of FGF-17 remain to be studied over a long period of time. The potential therapeutic mechanisms of FGF-17 are shown in Figure 2.

### 2.2. Endocrine fibroblast growth factors

FGF-19, FGF-21, and FGF-23 are members of the same subgroup, have low affinity for HS, and require the activation of corresponding receptors by binding to the KL transmembrane protein, which causes them to diffuse into the bloodstream and exert hormone-like regulatory effects readily. Therefore, they are also known as endocrine-FGFs. FGF-19 from intestinal epithelial cells and hepatocytes could restrain the synthesis of primary bile acids and alter the ratio of primary to secondary bile acids, thus indirectly regulating lipid metabolism and bile acid signaling (Owen et al., 2015; Al-Aqil et al., 2018). FGF-21, produced mainly by hepatocytes and adipocytes, is a vital regulator for maintaining the homeostasis of lipid, glucose, and energy metabolism (Kharitonenkov et al., 2005; Owen et al., 2015). FGF-23, synthesized in osteoblasts and osteocytes, is highly expressed in the kidney and parathyroid glands and regulates the dynamic balance of phosphate, calcium, and vitamin D metabolism (Cunningham et al., 2011; Vervloet, 2019). The above three FGFs have been shown to affect metabolism in the CNS and have a relationship with cognitive disorders and dementia (Hsuchou et al., 2013; Hensel et al., 2016; Li, 2019; Jiang et al., 2020).

#### 2.2.1. FGF-15/19

Researchers often refer to FGF-19 as FGF15/19, since FGF-19 in both humans and rats is expressed in mice as the homologous protein FGF-15. This protein is synthesized primarily in the enterocyte at the end of the ileum via the farnesoid X receptor (FXR) -related pathway and is expressed in the small intestine, liver, gallbladder, kidney, brain, and other tissues (Rysz et al., 2015). In tissues with rich β-KL, for example, the liver, FGF-15/19 binds to β-Kloth and activates FGFR-1, FGFR-2, FGFR-3, and FGFR-4, whereas, in tissues relatively deficient in β-KL like the brain, FGF-15/19 only binds to FGFR-4 (Nakamura et al., 2011). FGF-15/19 could stimulate the development of the heart and brain in the embryonic stage, regulate the production and circulation of bile acids (BAs) and promote glucolipid metabolism mainly in the liver at maturity; recently, it has also been found to have an insulin-like effect in the CNS, with the potential to tune sleep, cognition, and sensory functions (Hsuchou et al., 2013; Giacomini et al., 2016).

Due to the significant neuromodulatory, protective and nutritional effects of insulin on the brain, insulin resistance in the brain might exacerbate Aβ deposition, tau hyperphosphorylation, and vascular inflammation, causing cognitive impairments in AD and VaD (Hotamisligil, 2006; Kellar and Craft, 2020). Hypothalamic–pituitary–adrenal (HPA) axis hyperfunction is prevalent in dementia and other cognitive disorders, which might be the reason for higher adrenocorticotropic hormone (ACTH) and glucocorticoid (GC) levels, resulting in insulin resistance. Perry et al. (2015) have shown that intracerebroventricular injection of exogenous FGF-19 may improve insulin sensitivity by reducing serum ACTH and GC levels through inhibition of the HPA axis. This effect may arise from the inhibition of agouti-related protein/neuropeptide-Y (AGRP/NPY) neurons and the activation of the ERK1/2 pathway in the hypothalamus (Marcelin et al., 2014; Liu S. et al., 2018). In addition, recent studies have identified a link between the HPA axis dysfunction and cognitive decline in MCI and AD (Csernansky et al., 2006; Canet et al., 2019). Researchers have observed that the HPA axis dysfunction might emerge in the early stages of cognitive deficits, leading to structural damage in the hippocampus and cortex and NVU destabilization through the elevation of cortisol and norepinephrine (NE) levels with concomitant glucocorticoid receptor disruption and neurotoxicity (Popp et al., 2015; Canet et al., 2019). This finding was supported by Csernansky et al. (2006), Lara et al. (2013), and Wang L. Y. et al. (2018). Thus, the FGF-15/19 pathway might become a novel therapeutic target to maintain HPA axis function for drug discovery.

Furthermore, FGF-15/19 could affect NVU homeostasis by modulating BA production and circulation. The FXR for BA is found in neurons and ASTs in the hypothalamus and hippocampus of both humans and rodents (Mano et al., 2004; Huang et al., 2016; He et al., 2021), while physiological concentrations of BA can penetrate the BBB and suppress hyperexcitability of the HPA axis by activating glucocorticoid receptors in the hypothalamus and protect NVU by activating the BDNF–TrkB pathway (McMillin et al., 2015; Mertens et al., 2017; Li et al., 2020). Brain-derived neurotrophic factor (BDNF) is widely distributed in the CNS, especially in the cerebral cortex and hippocampus, with a significant role in the survival and maintenance of neurons. BDNF regulates synaptic plasticity via the autocrine pathway and modulates the presynaptic gamma-aminobutyric acid system via the paracrine pathway. BAs regulated by FGF-15/19 could enter hippocampal astrocytes and bind to FXR binding to promote BDNF synthesis, activate TrkB/PI3K/AKT pathway and TrkB/MAPK/ERK pathway, thus promoting hippocampal neurogenesis. Al-Aqil et al. (2018) observed that a high dosage of GC could elevate BA levels and downregulate FGF-15 expression in the serum and liver of mice. A recent metabolomics study identified that the serum concentrations of cholic acid (CA) and lithocholic acid (LCA) were significantly lower in patients with AD than in normal individuals (Pan et al., 2017). In contrast, the levels of CA, lithocholic acid, deoxycholic acid (DCA), chenodeoxycholic acid (CDCA), taurodeoxycholic acid (TDCA), and glycinodeoxycholic acid (GDCA) in serum, cerebrospinal fluid, and brain tissue have increased observably (MahmoudianDehkordi et al., 2019; Baloni et al., 2020). Changes in the ratio of secondary to primary BA have also been shown to be in close correlation with cognitive decline. For instance, the proportion changes of GDCA to CA shows a positive relationship with the amount of Aβ deposition in the cerebrospinal fluid in animal models of AD, while the proportion of TDCA and GDCA to CA is negatively correlated with glucose levels in the prefrontal cortex and hippocampus volume (MahmoudianDehkordi et al., 2019; Nho et al., 2019). Meanwhile, mounting evidence suggests that anti-dementia effects could be achieved with some common medicines used to treat gallstones, sclerosing cholangitis, sloughy hepatitis, and other cholestatic diseases. For example, tauroursodeoxycholic acid (TUDCA) has been insulin resistance shown to possess antidepressant efficacy and effectively inhibit Aβ deposition in AD (Lo et al., 2013; Lu et al., 2018; Cheng et al., 2019); Additionally, obeticholic acid, a synthetic FXR agonist that was developed based on the structure of CDCA, has been shown to enhance memory and cognition by maintaining BBB permeability and retarding neuronal degeneration (Gee et al., 2022). These findings suggest that modulation of BA-related pathways has the potential to be a new approach to improve mood and cognitive function and defer neuronal degeneration. In physiological conditions, FGF-15/19 activates the ERK1/2 pathway to inhibit cholesterol 7α-hydroxylase from preventing BA synthesis in order to bring BA concentration into equilibrium. In cases of dementia, disrupted endogenous glucocorticoids in the serum or disordered gut microbiota could restrict FGF-15/19 synthesis following FXR activation and counterbalance the inhibitory effect of FGF-15/19 on BA, thereby perturbing the balance of blood and tissue concentrations of BA. As a result, excessive BA would enter the CNS through circulation and cross the BBB, producing cytotoxicity, lysing the membranes of neuronal and endothelial cells, further disrupting NVU homeostasis. The following would aggravate the structural damage or lead to dysfunction in the hippocampus, hypothalamus, and other key regions, which would create a vicious cycle of cognitive impairment and morphological damage. Thus, FGF-15/19 may have an indirect regulatory role in the pathological changes seen in dementia by regulating glucose metabolism, HPA axis function, and BA homeostasis in critical brain regions. Although there is still a lack of direct evidence for changes in FGF-15/19 levels in dementia or other cognitive disorders, the above studies suggest a promising new therapeutic method for modulating BA synthesis, metabolism, and component ratios through the FGF-15/19 pathway to improve cognitive function. The potential therapeutic mechanisms of FGF-15/19 are shown in Figure 2.

#### 2.2.2. FGF-21

Fibroblast growth factor 21 (FGF-21) is primarily synthesized in the liver and adipose tissue, which is distributed throughout the bone, muscle, heart, kidney, and brain. FGF-21 is regulated by peroxisome proliferator-activated receptor-α (PPAR-α) and binds mainly to PPAR-γ, FGFR-1, and other receptors (Kuroda et al., 2017). FGF-21 has been proven to enhance glucose tolerance and insulin sensitivity, inhibit lipid synthesis and exert anti-inflammatory, antioxidant, and anti-apoptotic effects (Hui et al., 2018; Dolegowska et al., 2019). It could also penetrate the BBB to bind to FGFR-1 in the CNS and exert potent neuroprotective effects (Kuroda et al., 2017; Jiang et al., 2020). Researchers have currently identified that FGF-21 exerts neuroprotective effects through the following five significant pathways. (1) Protection of the BBB integrity: the FGF-21/β-KL/FGFR-1 pathway is automatically activated after cerebral microvascular injury or focal ischemia to mitigate neural and vascular endothelial damage. In addition, FGF-21 protects BBB integrity through the activation of PPAR-γ/PI3K/AKT/Rac1 pathway in cerebral microvascular endothelial cells and upregulation of tight junction proteins and adherent junction proteins expressions (Chen et al., 2018; Jiang et al., 2020). (2) Inhibition of neuroinflammation: FGF-21 could inhibit the pro-inflammatory phenotype of MG and NF-κB signaling pathway by suppressing the expression of pro-inflammatory factors such as IL-1β, IL-6, and TNF-α, in a way that nerve cells are protected from the damage caused by inflammation (Wang et al., 2020). (3) Promotion of neurogenesis: FGF-21 could activate PI3K/Caspase-3 signaling pathway and alleviate the apoptosis of neuronal cells (Zheng et al., 2019). In addition, FGF-21 may enhance hippocampal synaptic plasticity, increase dendritic spine density, promote restoration of mitochondrial function in the brain tissue, and inhibit apoptosis (Sa-Nguanmoo et al., 2016). Furthermore, since OPCs are widely distributed in the CNS, OLs differentiated from OPCs could be a crucial link between neural regeneration and myelin restoration. More specially, Kuroda et al. (2017) showed that the regulation of OPCs proliferation and differentiation by the FGF-21/β-KL/FGFR-1 pathway would be beneficial for neuroprotection. (4) Neurotransmitter regulation: FGF-21 could activate the HPA axis through the ERK/CREB pathway and induce the expression of corticotropin-releasing hormone (CRH) and ACTH, thereby regulating the secretion of serum corticosterone (Kuroda et al., 2017).

Researchers have gradually identified a close link between lipid metabolism disorders and dementia, especially AD. Disturbed lipid metabolism leads to aberrant levels and types of lipids such as fats, cholesterol, fatty acids, lipoproteins, and phospholipids. These abnormal changes affect the gut microbiota, brain-gut peptides, and neurotransmitter signaling and cause BBB disruption, mitochondrial dysfunction, oxidative stress, and inflammation, which eventually combine to cause a decline in synaptic plasticity and cognitive impairment (Kao et al., 2020). Lipidomics is a novel technique for researching the mechanism of lipid metabolism, which Akyol et al. (2021) have utilized to compare the biochemical profiles of brain tissues from patients with different degrees of AD. They found that different subgenera of lipids in AD were significantly disturbed, including neutral lipids, glycerolipids, glycerophospholipids, and sphingolipids (Akyol et al., 2021).

Interestingly, serum FGF-21 levels were reduced in both animal models and patients of AD but increased following cognitive improvement (Tournissac et al., 2019; Conte et al., 2021). Recombinant human FGF-21 could reduce the concentrations of total cholesterol, low-density lipoprotein, and high-density lipoprotein, inhibit neuroinflammation, and correct cognitive decline in cognitive impairment caused by hyperlipidaemia (Wang Q. et al., 2018). This result may be attributed to the capacity of FGF21 to regulate the lipolytic signaling pathway, or insulin signaling pathway, in hepatocytes (Gimeno and Moller, 2014). Researchers also found that the administration of exogenous FGF-21 suppressed the expression of β-site amyloid precursor protein cleaving enzyme1 (Bace1), reduced Aβ deposition, and improved manifestations of dementia by inhibiting neuroinflammation through the TLR4/NF-κB signaling pathway, which could also restrain apoptosis via the MAPK signaling pathway (Amiri et al., 2018; Chen S. et al., 2019; Taliyan et al., 2019). These suggest a correlation between FGF-21 and AD, which makes it a potential biomarker for exploring new biomarkers, developing new drug targets, and providing new directions for in-depth exploration of AD metabolic mechanisms. Besides, since FGF21 could improve neuronal metabolism and energy supply in the CNS, enhance neuronal plasticity, and repair cerebrovascular endothelium to relieve symptoms of cognitive impairment, developing new drugs based on FGF-21 might be the most suitable choice to explore a new therapy for VaD. The potential therapeutic mechanisms of FGF-21 are shown in Figure 2.

#### 2.2.3. FGF-23

FGF-23 is a bone-released endocrine growth factor and a member of the FGF-19 subgroup. FGF-23 is synthesized by osteoblasts and osteoclasts that are distributed primarily in the kidney, cortex, hippocampus, hypothalamus, thyroid gland, and bone, but also to a small degree in the spleen, liver, and other tissues (Hensel et al., 2016; Kuro-O, 2021; Ursem et al., 2021). FGF-23 primarily binds to α-KL/FGFR-1 to exert physiological effects, including regulating phosphate homeostasis and glucose metabolism, promoting neurogenesis, and maintaining emotional and cognitive functions (Beenken and Mohammadi, 2009; Kuro-O, 2021). FGF-23 overexpression in the serum may induce impaired hippocampal long-time potentiation (LTP) and reduce hippocampal adenosine-triphosphate (ATP) content with cognitive and memory decline, especially in people with chronic kidney disease (Liu et al., 2011; Drew et al., 2014; Drew and Weiner, 2014). Although Zhu et al. (2018) arrived at the opposite conclusion as they observed no significant differences between the levels of serum FGF-23 in individuals with mild cognitive impairment and healthy individuals, however, they assayed FGF-23 in a different way compared to previous experiments, which may explain the discrepancy in the results. The difference might also be attributed to the fact that FGF-23, like FGF-21, is affected by sex, and it is also possible that FGF-23 levels in the cerebrospinal fluid and blood are different. FGF-23 knockout mice also exhibited cognitive impairment in several *in vivo* studies, which may be associated with dysregulation of phosphate homeostasis and cytotoxicity (Laszczyk et al., 2019). As for the *in vitro* studies, the FGF-23/α-KL/FGFR-1 pathway was shown to increase hippocampal neuronal synaptic density but inhibits neuronal ramification via activating the downstream Akt signaling pathway, leading to memory deficits (Hensel et al., 2016; Zhu et al., 2018). These difference between animal and cellular experiments suggest that FGF-23 may have bidirectional modulatory effects on cognitive function.

On the other hand, intracranial atherosclerosis may trigger endothelial injury in blood vessels, induce neuroinflammation, and thus damage the structure and function of key brain regions where emotion and cognition are regulated, such as the hippocampus (Castello et al., 2022). In the vascular system, FGF-23 could bind directly to FGFR-2 or FGFR-3 without α-KL to induce vascular calcification (Vervloet, 2019). Thus, FGF-23 is highly associated with atherosclerosis and is a significant risk factor for atherosclerosis and stroke (Fakhri et al., 2014; Chang et al., 2020; Zheng et al., 2020). In other words, FGF-23 may indirectly trigger cognitive impairment by aggravating cerebrovascular damage. The potential therapeutic mechanisms of FGF-23 are shown in Figure 2.

## 3. Fibroblast growth factor receptors in cognitive disorders and dementia

The four main fractions of the transmembrane receptor tyrosine kinase FGFR, including FGFR-1, FGFR-2, FGFR-3, and FGFR-4, can mediate the signaling of FGFs via HS or KL-dependent pathways. Although all FGFRs are widely distributed in the CNS, existing evidence suggests that the main receptor that has been observed to change significantly in cognitive disorders is FGFR-1 (Goswami et al., 2013).

### 3.1. FGFRs

FGFR-1 is predominantly expressed in the hippocampus, and mediates downstream pathways by inhibiting neuroinflammation and maintaining LTP to protect learning and cognitive abilities (Rajendran et al., 2021). FGF-2, FGF-9, and FGF-22 can bind directly to FGFR-1 in the CNS. For example, the BBB protective effect on the BBB exhibited by FGF-2 is achieved by activating FGFR-1 (Lin et al., 2018), whereas the involvement of β-KL is required for binding FGF-21 and FGF-23 to FGFR-1, as mentioned above. Alternatively, dramatic AHN restriction, diminished amplitude of LTP, and hypomnesia could be observed in FGFR-1 knockout mice, and these changes were reversed with FGFR-1 agonists (Zhao et al., 2007; Pereda-Pérez et al., 2019). A compensatory increase in FGFR-3 expression can be observed in ASTs in the vicinity of “senile plaques” in AD patients (Ferrer and Martí, 1998). Moreover, FGF-2 binding to FGFR-3 could activate the anti-inflammatory phenotype of MG, inhibit excitatory toxicity, and exert neuroprotective effects through regulation of the ERK1/2 signaling pathway (Noda et al., 2014). Although the expression of FGFR-4 is low in the CNS and mainly concentrated in specific brain areas, especially the hypothalamus, FGF-15/19 could only inhibit the HPA axis and exert insulin-like effects by specifically binding to FGFR-4 due to the relatively low number of β-KL in the hypothalamus. Experiments by Ryan et al. (2013) showed that FGF-15/19/β-KL/FGFR-4 in the hypothalamus plays an integral role in regulating glucose metabolism throughout the body. As previously mentioned, abnormal glucose metabolism is strongly associated with the development of cognitive impairment and dementia. FGFRs demonstrate high homology and overlapping recognizability, which means there is no corresponding relationship between FGFRs and FGFs. Thus, it is difficult to study the changes in FGFRs. However, the future progressive exploration of the correspondence between FGFs and FGFRs and their downstream pathways will have profound implications for the study and application of the FGFs/FGFRs system in the field of neuroscience.

### 3.2. Co-receptor KL protein

Endocrine FGFs have a poor affinity to HS, and all have to form different homodimers by binding the co-receptor KL protein to the corresponding FGFR to activate the downstream pathways, such as FGF-19/β-KL/FGFR-4, FGF-21/β-KL/FGFR-1, or FGF-23/α-KL/FGFR-1. Subsequently, endocrine FGFs exert beneficial cognitive effects by inhibiting the HPA axis activation, suppressing neuroinflammation, facilitating BA metabolism, enhancing insulin sensitivity, and promoting neurogenesis. KL is divided into three categories according to the protein-coding genes, namely low molecular weight α-KL, high molecular weight β-KL, and γ-KL (Massó et al., 2015; Zhou et al., 2015). The α-KL can be further divided into transmembrane (m-KL), free, and secretory type, with the latter two referred to as soluble KL (s-KL), which is one of the hot spots in current dementia research. Researchers have only observed γ-KL in brown adipose tissue and eyeballs and have not yet found an association with the FGFs/FGFRs system (Zhang et al., 2017; Ma et al., 2021). Although much evidence suggests that α-KL has essential effects on cognition and memory, these studies have focused on s-KL rather than m-KL as a ligand for FGF-23 (Cararo-Lopes et al., 2017; Li et al., 2017; Zhao et al., 2020; Tank et al., 2021). These two α-KL have different structural features and physiological roles. However, recent studies have also identified, that hydrolyzed m-KL is a significant source of s-KL (Chen et al., 2007). β-KL protein is mainly distributed in the liver, gallbladder, kidney, brain, and other tissues, synthesized by ependymal cells in the hippocampus in CNS, and excreted into the cerebrospinal fluid, where it can exert antioxidant, anti-inflammatory, and neuroprotective effects (Paroni et al., 2019). These effects may be attributed directly to inhibiting neuroinflammation, reducing oxidative stress, and correcting vascular endothelial dysfunction or indirectly by activating pathways related to endocrine FGFs (Gold et al., 2013).

## 4. Discussion

FGFs and their receptors constitute a complex network of endocrine functions, and the dynamic balance between FGFs and their receptors may be a critical link that our understanding of cognitive regulation. When the FGFs/FGFRs system is out of balance, pathological changes such as disturbance of glucose metabolism disorder, neuroinflammation, hyperfunction of the HPA axis, disruption of the BBB, reduced neuroplasticity, inhibition of neurogenesis, and apoptosis may occur in the body, which in turn can affect the structure and function of the cerebral cortex, hippocampus, hypothalamus, pituitary gland, and other tissues, resulting in cognitive decline. Therefore, maintaining the stability of the FGF-FGFR system might have the potential to be a new therapeutic strategy for retarding neurodegeneration and improving cognitive function. Endocrine FGFs could be distributed through the blood circulation to multiple organs throughout the body, so they can exert a comprehensive neuroprotective effect by intervening in multiple systems. Besides, The physiological characteristics of endocrine FGFs allow them to penetrate the BBB to act on the brain parenchyma and exert neuroprotective effects easier than paracrine FGFs, including FGF-1, FGF-2, and FGF-9, via multiple metabolic pathways.

**Figure 3:**
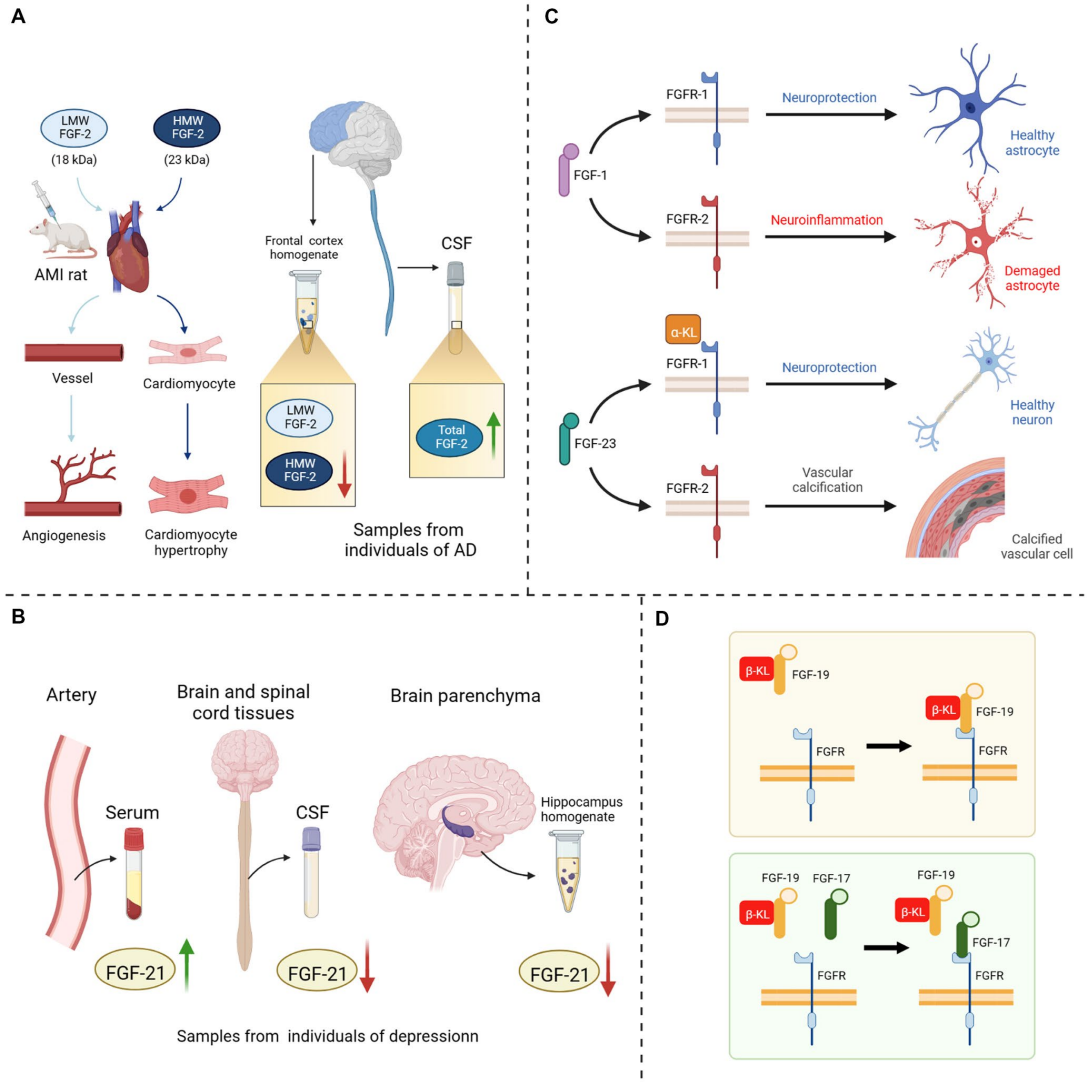
Four potential explanations for the hampered FGF in-depth study. **(A)** Different isoforms of FGF-2: injection of HMW FGF-2 into AMI rats could induce cardiomyocyte hypertrophy, whereas injection of LMW FGF-2 only promoted angiogenesis in rat heart; A decrease in HMW FGF-2 expression could be detected in the anterior cortical homogenate of AD patients, while the expression level of LMW FGF-2 remained unchanged, and the level of total FGF-2 in the CSF was significantly increased. **(B)** Inhomogeneous space distribution of FGF21: the expression level of FGF-21 was elevated in the serum of depressed patients, while decreased in both CSF and hippocampus homogenate. **(C)** Correspondence between FGFs and FGFRs: when FGF-1 bound to FGFR-1, it could exert neuroprotective effects, while binding to FGFR-2 could provoke neuroinflammation; The binding of FGF-23 to α-KL/FGFR-1 protects neuronal cells, on the contrary it directly binds to FGFR-2 to induce vascular calcification. **(D)** Competitive inhibition between FGF17 and FGF19: FGF-17 could bind to the β-KL/FGFR-2 homodimer and shown antagonism against FGF-19. LMW, light molecular weight; HMW, high molecular weight; AMI, acute myocardial infarction; CSF, cerebrospinal fluid; AD, Alzheimer’s disease; FGF, fibroblast growth factor; FGFR, fibroblast growth factor receptor; α/β-KL, α/β-klotho protein.

In recent years, FGFs-based drugs have been widely used in the clinical treatment of wound healing, osteoarthritis, malignancies, hepatitis, diabetes, cardiovascular disease and cerebrovascular disease (Zhang and Li, 2016; Sanyal et al., 2019; Abou-Alfa et al., 2020; Chen K. et al., 2022; Subbiah et al., 2022). Although the relevance of the FGF-FGFR system to neurodegenerative diseases has been identified, the application of FGF-based drugs remains *in vivo* and *in vivo* experiments which are still far from clinical application for treating cognitive disorders. Moreover, the use of FGFs as biomarkers for detecting neurodegenerative diseases is also limited by many factors. Although researchers are still unable to elucidate the specific mechanisms of the FGFs/FGFRs system, they have noted the trends and therapeutic potential of multiple FGFs in various diseases and have discovered or developed drugs, such as synthetic human recombinant FGFs (Bogousslavsky et al., 2002), synthetic analogs of FGFs (Sanyal et al., 2019; Ardizzone et al., 2022), molecular inhibitors or agonists of FGFs/FGFRs (Bahleda et al., 2020; van Brummelen et al., 2020; Subbiah et al., 2022), or extracts of natural herbal medicines (Hong et al., 2016; Yuan et al., 2019; Cheng et al., 2021; Wang et al., 2021) that could effectively modulate this system. In the current literature review, we found that the difficulties faced by previous studies on the application of FGFs/FGFRs systems in treatment and diagnose mainly focus on the following four aspects (Figure 3): (1) Different FGFs isoforms: some FGFs have different molecular weight isoforms with different physiological effects, but their individual microscopic expression changes cannot be reflected by the overall changes, for example, High molecular weight (HMW) FGF-2 and Light molecular weight (LMW) FGF-2 (Chlebova et al., 2009; Katsouri et al., 2015; Chen X. et al., 2019). (2) Inhomogeneous space distribution of FGFs: the spatial distribution of FGFs in serum, cerebrospinal fluid and brain parenchyma is inhomogeneous, because some FGFs cannot completely penetrate the BBB. The following may obscure particular properties of FGFs in various tissues under pathological state, and render the statistical assessment their heterogeneity inaccurate (Katsouri et al., 2015; Liu et al., 2017; Chang et al., 2018; Tennakoon et al., 2022). (3) Correspondence between FGFs and FGFRs: single FGF could bind to different FGFRs in different microenvironments and regulate different signaling pathways, thus exerting distinct physiological or pathological effects, due to the overlapping recognition of FGFRs and discrepancy in expression sites (Lee et al., 2011; Noda et al., 2014; Lin et al., 2018; Vervloet, 2019). (4) Competitive inhibition between FGFs: FGFs are highly homologous, and subtypes with similar physiological structures may compete with one another for the same receptors, thereby inhibiting the regulation of downstream signaling pathways such as FGF-17 and FGF-15/19 (Liu S. et al., 2018), FGF-2 and FGF-9 (Aurbach et al., 2015).

## 5. Conclusion

In summary, despite the induced cell proliferation, survival and differentiation, angiogenesis promotion, inflammation inhibition, immune and metabolic modulation, and antioxidant effects possessed by the FGF/FGFR system have been applied in the treatment of tumors, trauma, and renal, hepatic, and cardiovascular diseases, there is still a substantial unexplored gap area, especially for the modulatory effects and diagnostic value of central nervous system diseases and psychiatric disorders. Considering the extensive range of actions and the many targets of the FGF/FGFR system intervention, we should also be aware of the potential risk associated with its long-term or high-dose use, such as adverse effects, resistance, and addiction, when used to treat cognitive or mood disorders. Here, we propose to conduct more clinical and basic studies in combination with new technologies to investigate the structural and functional characteristics of the FGFs/FGFRs system and its specific mechanism in cognitive disorders and dementia to find new biomarkers and therapeutic approaches for these diseases.

## Author contributions

WZ and TZ conceived the topic and drafted the manuscript. YJ helped WZ to draw the figures. SH, MX, and JP revised the draft. All authors contributed to the article and approved the submitted version.

## Funding

This research was supported by National Natural Science Foundation of China (NSFC-81573790, NSFC-81603443, and NSFC-82204922) and the Fundamental Research Funds for the Central public welfare research institutes of China (no. ZZ15-XY-LCQ-04).

## Conflict of interest

The authors declare that the research was conducted in the absence of any commercial or financial relationships that could be construed as a potential conflict of interest.

## Publisher’s note

All claims expressed in this article are solely those of the authors and do not necessarily represent those of their affiliated organizations, or those of the publisher, the editors and the reviewers. Any product that may be evaluated in this article, or claim that may be made by its manufacturer, is not guaranteed or endorsed by the publisher.

## Figures and Tables

**Table:** 

ACTH	Adrenocorticotropic hormone
AD	Alzheimer’s disease
ADPN	Adiponectin
aFGF	Acidic fibroblast growth factor
AGRP/NPY	Agouti-related protein/neuropeptide-Y
AHN	Adult hippocampal neurogenesis
AKT	Protein kinase B
AMI	Acute myocardial infarction
AMPK	Adenosine monophosphate activated protein kinase
AST	Astrocytes
ATP	Adenosine-triphosphate
Aβ	Amyloid-β
BA	Bile acid
BBB	Blood–brain barrier
BDNF	Brain-derived neurotrophic factor
bFGF	Basic fibroblast growth factor
CA	Cholic acid
CDCA	Chenodesoxycholic acid
CNS	Central nervous system
CREB	cAMP-response element binding protein
CSF	Cerebral spinal fluid
DCA	Deoxycholic acid
ERK	Extracellular signal-regulated kinase
FGF-1	Fibroblast growth factor-1
FGF-2	Fibroblast growth factor-2
FGF-9	Fibroblast growth factor-9
FGF-15/19	Fibroblast growth factor-15/19
FGF-17	Fibroblast growth factor-17
FGF-21	Fibroblast growth factor-21
FGF-23	Fibroblast growth factor-23
FGFR-1	Fibroblast growth factor receptor-1
FGFR-2	Fibroblast growth factor receptor-2
FGFR-3	Fibroblast growth factor receptor-3
FGFR-4	Fibroblast growth factor receptor-4
FGFRs	Fibroblast growth factor receptors
FGFs	Fibroblast growth factors
FXR	Farnesoid X receptor
GAP-43	Growth association protein-43
GC	Glucocorticoid
GDCA	Glycinodeoxycholic acid
GR	Glutathione reductase
GSK-3β	Glycogen synthase kinase-3β
HD	Huntington’s disease
HMW	High molecular weight
HPA	Hypothalamic–pituitary–adrenal
HS	Heparan sulfate
HXK2	Hexokinase 2
IL-6	Interleukin-6
IL-β	Interleukin-1β
LCA	Lithocholic acid
LMW	Light molecular weight
LTP	Long-time potentiation
MAP-2	Microtubule-associated protein-2
MAPK	Mitogen-activated protein kinase
MCI	Mild cognitive impairment
MG	Microglial
m-KL	Membrane Klotho
NE	Norepinephrine
NF-κB	Nuclear factor kappa-B
NMDAR	N-methyl-D-aspartic acid receptor
Nrf-2	Nuclear factor erythroid-like 2
NVU	Neurovascular unit
OL	Oligodendrocyte
OPC	Oligodendrocyte precursor cell
PGC-1α	Peroxisome proliferator-activated receptors-γ coactivator 1-α
PI3K	Phosphatidylinositol-3-hydroxy kinase
PPAR-α	Peroxisome proliferator-activated receptor-α
PPAR-γ	Peroxisome proliferator-activated receptor-γ
Rac1	Rac family small GTPase 1
RhoA	Ras homolog family member A
SIR1	Sirtuin1
s-KL	Soluble Klotho
SOD2	Superoxide dismutase 2
TDCA	Taurodeoxycholic acid
TLR4	Toll like receptor-4
TNF-α	Tumor growth factor-α
TrkB	Tyrosine kinase receptor B
TUDCA	Tauroursodeoxycholic acid
VaD	Vascular dementia
VEGF	Vascular endothelial growth factor
WMH	White matter hyperintensities
α/β/γ-KL	α/β/γ-Klotho protein
γ-GCS	Gamma-glutamylcysteine synthetase
